# Global population structure of Shiga toxin-producing Escherichia coli O103:H2 and the variation in their major virulence factor-encoding genetic elements

**DOI:** 10.1099/mgen.0.001625

**Published:** 2026-01-23

**Authors:** Itsuki Taniguchi, Yo Morimoto, Yoko Kimura, Junji Seto, Yuko Kawai, Tomoko Kitahashi, Junko Aoki, Katsuya Terai, Toshihiko Furuta, Yuki Wakabayashi, Sumiko Tanabe, Mitsuhiro Hamasaki, Yuri Abe, Mari Sasaki, Hiroshi Narimatsu, Eiji Yokoyama, Sunao Iyoda, Tetsuya Hayashi, Keiji Nakamura

**Affiliations:** 1 Graduate School of Medical Sciences, Kyushu University, Fukuoka, Japan; 2 Hokkaido Institute of Public Health, Sapporo, Japan; 3 Miyagi Prefectural Institute of Public Health and Environment, Sendai, Japan; 4 Yamagata Prefectural Institute of Public Health, Yamagata, Japan; 5 Gunma Prefectural Institute of Public Health and Environmental Sciences, Gunma, Japan; 6 Chiba City Institute of Health and Environment, Chiba, Japan; 7 Niigata Prefectural Institute of Public Health and Environmental Sciences, Niigata, Japan; 8 Shizuoka Institute of Environment and Hygiene, Shizuoka, Japan; 9 Hamamatsu City Health Environment Research Center, Shizuoka, Japan; 10 Osaka Institute of Public Health, Osaka, Japan; 11 Nara Prefecture Institute of Health, Nara, Japan; 12 Fukuoka Institute of Health and Environmental Sciences, Fukuoka, Japan; 13 Fukuoka City Institute of Health and Environment, Fukuoka, Japan; 14 Oita Prefectural Institute of Health and Environment, Oita, Japan; 15 Chiba Prefectural Institute of Public Health, Chiba, Japan; 16 National Institute of Infectious Diseases, Tokyo, Japan

**Keywords:** Shiga toxin-producing *Escherichia coli*, O103:H2, comparative genomics, Shiga toxin (Stx) phage, locus of enterocyte effacement (LEE), Shiga toxin-producing *Escherichia coli* (STEC) virulence plasmid

## Abstract

Shiga toxin (Stx)-producing *Escherichia coli* (STEC) is a major cause of serious gastrointestinal illness, including diarrhoea, haemorrhagic colitis and life-threatening haemolytic-uraemic syndrome. Although O157:H7 STEC strains are the most prevalent, the incidence of STEC infections caused by several other serotypes has recently increased. O103:H2 STEC is one of these major non-O157 STEC strains, but systematic whole-genome sequence (WGS) analyses have not yet been conducted. To gain a global phylogenetic overview of O103:H2 STEC based on WGSs, we analysed 2,701 WGSs of O103:H2 strains, including 193 sequenced in this study. Sequence type (ST)-based classification divided the O103:H2 strains into three distinct *E. coli* lineages. As the virulence marker genes of typical STECs (*stx*, *eae* and *ehxA*) were found only in the major O103:H2 lineage (*n*=2,658) comprising ST17 and its single- and double-locus variants, we performed a global phylogenetic analysis of the major lineage. This analysis revealed that this lineage was divided into five clades (C1–C5) and that C1 was the ancestral clade, C2 and C3 emerged from C1 and C4 and C5 emerged from C3. While *stx2* genes were sporadically distributed in limited STEC O103:H2 strains, *stx1a*, *eae* and *ehxA* were highly conserved throughout the entire STEC O103:H2 lineage. However, through a detailed comparison of seven closed genomes of STEC strains, covering the five clades and including four obtained in this study, we found marked variation in the genetic elements encoding the virulence genes (Stx1a phage, the locus of enterocyte effacement (LEE) and the virulence plasmid), such as rearrangement in the LEE accessory region, a shift in the integration sites of the Stx1a phage due to the replacement of the integrase gene-containing genomic segments, the replacement of the virulence plasmid and the gain and loss of virulence-related genes in the virulence plasmid. Overall, this study highlights the current global population structure of O103:H2 strains and provides evolutionary insights into the variation in virulence determinants within STEC O103:H2, which is relatively understudied among the major STEC lineages.

**Impact Statement**

Shiga toxin (Stx)-producing *Escherichia coli* (STEC) are important foodborne pathogens that cause not only mild diarrhoea but also severe haemorrhagic colitis and life-threatening haemolytic uraemic syndrome. Serotype O157:H7 is the most predominant STEC, but infections by several non-O157 STEC strains, including O103:H2 STEC, have recently increased worldwide, which was partly because of a diagnostic method change. However, owing to the lack of systematic whole-genome sequence (WGS) analyses, the population structure and genomic diversity of O103:H2 STEC are unknown. Here, we produced WGSs of 193 Japanese strains, including 4 closed genomes, to expand the genomic information resources for O103:H2 and performed a global WGS analysis of 2,701 O103:H2 strains and a detailed comparison of the closed genomes. Through these analyses, we identified three distinct O103:H2 lineages and found that the virulence marker genes for typical STECs were found only in the major lineage. Although the STEC virulence marker genes were highly conserved in the major O103:H2 lineage, the genetic elements (Stx-transducing phage, the locus of enterocyte effacement and the virulence plasmid) encoding these genes exhibited notable variations. Thus, this study highlights the current global population structure of O103:H2 strains and the variation in the major virulence determinants within this relatively understudied STEC.

## Data Summary

The finished genomes of four strains and the Illumina read sequences of 203 O103:H2 strains obtained in this study have been deposited in DDBJ/EMBL/GenBank under BioProject accession numbers starting from PRJDB19807 (https://www.ncbi.nlm.nih.gov/bioproject, see Table S1, available in the online Supplementary Material, for the accession numbers of each strain).

## Introduction

Shiga toxin (Stx)-producing *Escherichia coli* (STEC) are foodborne pathogens that cause a range of diseases, ranging from mild enteritis to severe haemorrhagic colitis and sometimes life-threatening complications, such as haemolytic uraemic syndrome [1,2]. The major virulence factors of STEC are Stxs encoded by prophages (Stx phages). Stxs are classified into Stx1 and Stx2, and both include multiple variants (Stx1a, Stx1c-Stx1e; Stx2a-Stx2o) [3,5]. Typical STECs responsible for human infections share the locus of enterocyte effacement (LEE), which encodes a type III secretion system (T3SS), with enteropathogenic *E. coli* (EPEC) [6], and more than 30 genes encoding effectors have been carried into STEC and EPEC by multiple prophages [7,9]. Therefore, EPEC strains are generally regarded as progenitors of typical STEC strains. In addition, most STEC strains carry a large virulence plasmid, which harbours an incFIB replicon [10] and encodes enterohaemolysin and other potential virulence factors.

Among various STEC serotypes, O157:H7 is most prevalent worldwide, but strains of other serotypes also cause outbreaks and sporadic cases of infection. O103:H2 is one of the major non-O157 STEC serotypes that causes severe diseases [11,13] and is frequently isolated from humans and cattle [14,16]. The majority of O103:H2 strains harbour *stx1a* alone, and isolates carrying *stx2a* are rarely found [14,17, 18]. While the *eae* and *ehxA* genes (the marker genes for LEE and virulence plasmids, respectively) are well conserved in O103:H2 strains, virulence plasmid-encoded genes other than *ehxA*, such as *katP* (catalase), *espP* (serine protease) and *etp* (a type II secretion system gene), are variably distributed [14,17].

Whole-genome sequence (WGS)-based phylogenetic analysis of O103:H2 at the global level has not been conducted. Recently, the WGS-based population structure of clonal complex 17 (CC17), which includes O103:H2 strains (more than half of a total of 420 isolates), has been published, but the analysis was limited to strains isolated in England and Wales [19]. In the present study, to analyse the global population structure and evolutionary history of STEC O103:H2, the major serotype within CC17, we collected and analysed 2,701 WGSs of O103:H2 strains, which included 193 WGSs determined in this study, and found that the O103:H2 strains can be classified into three distinct *E. coli* lineages and that the STEC virulence genes are distributed in the major lineage. We therefore further performed a WGS-based phylogenetic analysis of the O103:H2 strains belonging to the major lineage (*n*=2,658) and in-depth analyses of the Stx phages, LEE and plasmids in seven closed genomes.

## Methods

### Bacterial strains

In this study, we sequenced 203 O103:H2 strains isolated in Japan, all of which were human isolates. Of these 203 sequences, 2 were not properly assembled and 8 were low-quality (completeness <99% or contamination >1%, as determined by CheckM [20]); thus, these sequences were excluded. Of the remaining 193 WGSs of Japanese strains, four were closed as described below. To collect O103:H2 WGS data available in public databases, we downloaded O103:H2 read or assembled sequence data from the National Center for Biotechnology Information (NCBI) database and O103:H2 assembled sequences from EnteroBase (*n*=3013, access date 28 February 2022). After confirming their serotypes using Abricate (https://github.com/tseemann/abricate) with the EcOH database [21] and excluding low-quality sequences with the same threshold as that described above, 2,508 genomes were included in the dataset. Thus, a total of 2,701 O103:H2 strains were analysed (listed in Tables 1 and S1). Their sequence types (STs) were determined by a blastn-based strategy using the mlst program v2.23.0 (https://github.com/tseemann/mlst) with Achtman’s scheme and the PubMLST database (https://pubmlst.org). Genomes whose STs were not precisely defined were reanalysed by a read mapping-based strategy using the SRST2 program v0.2.0 [22] with the default parameters. Relationships between the identified STs were analysed via the MSTree V2 algorithm packaged in the GrapeTree program [23], and a minimum spanning tree (MST) based on the STs was visualized using the same program.

**Table 1.** The O103:H2 strain set analysed in this study

Country	Source				Total
	Human	Animals	Foods/environments	No information	
Japan	197	3	0	0	200
USA/Canada	1,814	296	100	10	2,220
UK	178	10	1	13	202
Other European countries*	20	26	3	1	50
Other countries†	6	3	0	0	9
No information	1	0	0	19	20
Total	2,216	338	104	43	2,701

*Denmark (*n*=15), Sweden (*n*=14), Germany (*n*=9), Belgium (*n*=4), Italy (*n*=3), Austria (*n*=1), Hungary (*n*=1), Ireland (*n*=1), Norway (*n*=1), Portugal (*n*=1).

†Argentina (*n*=3), Kenya (*n*=2), Australia (*n*=2), New Zealand (*n*=1), China (*n*=1).

### Genome sequencing, assembly and annotation

The purification of genomic DNA, preparation of sequencing libraries, Illumina MiSeq sequencing and sequence assembly were performed as previously described [24]. The average sequencing depth and number of scaffolds were 47×‒378× and 187‒850, respectively. Four strains (129, PV16-126, CEC12044 and 20151001) were additionally sequenced using MinION with R10.4.1(129 and PV16-126) or R9.4.1 (CEC12044 and 20151001) flow cells [Oxford Nanopore Technologies (ONT)] for 72 h (129 and PV16-126) or 96 h (CEC12044 and 20151001). Read data in fastq format were generated using MinKNOW v23.4.8 and Guppy v6.5.7 (129 and PV16-126) or MinKNOW v3.1.13 and qcat v1.1.0 (CEC12044 and 20151001). These reads were trimmed and filtered using the following programme and parameters: trimming using Porechop (v0.2.4) [25] and filtering over 2 kb at a quality score (Q score) > 8 using NanoFilt (v2.8.0) [26], with the option of trimming 100 bp from the start of the read in strains 129 and PV16-126, or filtering over 2 kb at a Q score >10 using NanoFilt (v2.3.0), with the option of trimming 100 bp from the start of the read in strains CEC12044 and 20151001. The trimmed and filtered ONT and Illumina reads of each strain were assembled using the MicroPIPE pipeline [27] for strains 129 and PV16-126 or Unicycler v0.4.7 [28] for strains CEC12044 and 20151001. A tandemly duplicated genome generated for a small plasmid (~8 kb) in strain 129 was manually corrected. The closed genomes were annotated using DFAST [29], followed by manual curation. GenomeMatcher (v3.0.8) [30] was used for genome sequence comparison and dot-plot analysis and to display the results. The presence of plasmid replicons was determined using PlasmidFinder v.2.1 [31] with default parameters.

### SNP detection and phylogenetic analysis

Phylogenetic analyses were performed for the O103:H2 strain set comprising strains belonging to ST17 (*n*=2,169) and its single-locus variants (SLVs) and double-locus variants (DLVs) (482 and 7, respectively) (Tables 2 and S1) using the O103:H2 strain 12009 as a reference with or without the O26:H11 strain 11368 [9]. For these analyses, SNP sites on prophage (PP)/integrative element (IE)/insertion sequence (IS)-free and recombination-free chromosome backbone sequences conserved in all analysed genomes were identified using MUMmer [32] and Gubbins [33] to construct maximum likelihood (ML) trees using RAxML as previously described [34]. For tree construction, strains were deduplicated if the chromosome backbone sequences were identical. Clustering analysis of O103:H2 strains was performed using Fastbaps v1.0.8 with ‘BAPS’ [35], and clades were defined at the first level of hierarchical clustering. ML trees were displayed using iTOL [36].

**Table 2.** STs and STEC/EPEC virulence genes of the O103:H2 strains

			*stx* genotype										*eae* subtype			*ehxA*	
ST17 and locus variants of ST17	No. of STs	No. of strains	1a	1a×2^*^	1c	1 a/2 a	1 a/2 c	1 a/2d	2a	2c	2d	Negative	Epsilon1	Beta1	Negative	Positive	Negative
ST17	1	2,169	2,061	0	0	32	2	14	13	1	1	45	2,137	1	31	2,066	103
SLVs	32	482	435	1	0	21	0	0	1	0	0	24	470	11	1	442	40
DLVs	6	7	4	0	0	0	0	0	0	0	0	3	4	3	0	3	4
4LV	5	35	0	0	1	0	0	0	0	0	0	34	0	0	35	0	35
6LV (ST1146)	1	8	0	0	0	0	0	0	0	0	0	8	0	0	8	0	8
Total	45	2,701	2,500	1	1	53	2	14	14	1	1	114	2,611	15	75	2511	190

*Two copies of *stx1a* genes.

4LV, quadruple-locus variant; 6LV, sextuple-locus variant.

### Analysis of the *stx*, *eae* and *ehxa* genes

The subtypes of *stx* and *eae* were determined by blastn as previously described [37], but with slightly different thresholds (>98% identity and >50% coverage for *stx* and >99% identity and >90% coverage for *eae*). Plasmid-encoded virulence genes (*ehxA*, *ecf1*, *stcE*, *katP*, *espP* and *efa1*) were also identified by a blastn search (>98% identity and >30% coverage), with the representative sequences of each gene used as references (listed in Table S2). As both *stx* and plasmid-encoded virulence genes were sometimes fragmented in draft genomes, we used a lower threshold than that applied to the *eae* gene.

## Results and discussion

### Strain set

We analysed a total of 2,701 O103:H2 genome-sequenced strains in this study. These strains included 193 Japanese strains sequenced in this study (all human isolates) and 2,508 strains whose genomes were obtained from public databases and were isolated in various geographical regions (Tables 1 and S1). North American (USA/Canada) strains represented 82 % of the strain set, followed by strains from the UK (*n*=202) and Japan (*n*=200). Although most strains (*n*=2,216) were human isolates, isolates from nonhuman animals (bovine, avian, swine and others; *n*=338) and foods/environments (beef, water, plant and others; *n*=104) were also included.

**Fig. 1.**
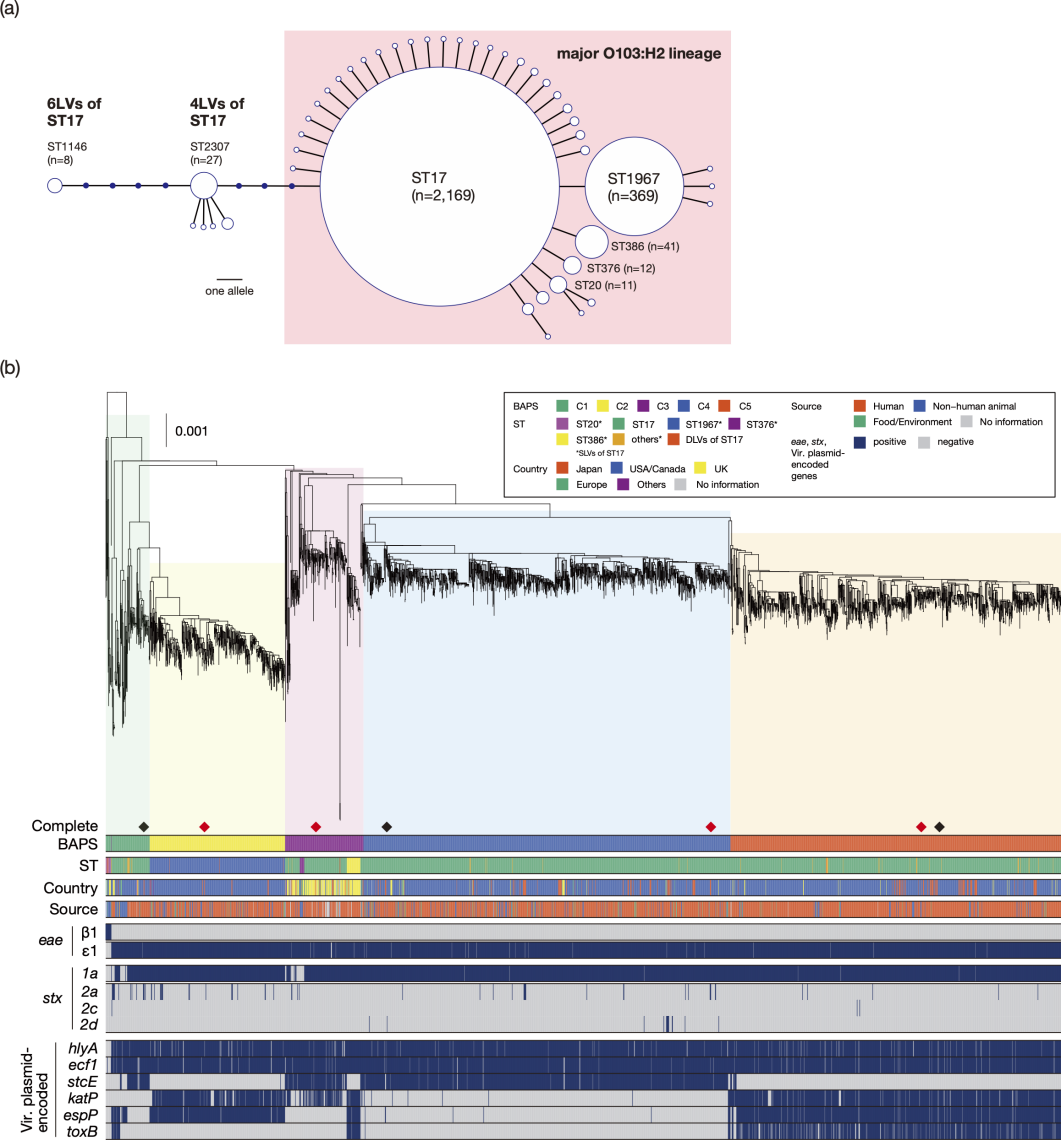
Relationships among the *E. coli* O103:H2 strains analysed in this study. (**a**) An MST displaying the population structure of 2701 O103:H2 strains based on allele sequence combinations of the *adk*, *fumC*, *gyrB*, *icd*, *mdh*, *purA* and *recA* genes. The sizes of the open circles representing each ST are scaled to the number of isolates belonging to each ST. Hypothetical STs not found in the strain set are represented by filled circles. The O103:H2 major lineage, to which ST17 and its SLVs and DLVs belong, is highlighted. (**b**) Phylogenetic relationships of 2,497 strains belonging to the major O103:H2 lineage. An ML tree constructed based on 29,938 SNPs identified on the PP/IE/IS-free and recombination-free chromosomal backbone (2,045,250 bp) is shown along with strain information on Fastbaps clades (BAPS), STs, isolation countries and sources, the presence/absence of *eae* (subtypes *β*1 and *ε*1), *stx* (*stx1a*, *stx2a*, *stx2c* and *stx2d*) and the STEC virulence plasmid (Vir. Plasmid)-encoded virulence-related genes. Strains belonging to ST20 and its SLVs are used as outliers according to the results shown in Fig. S1. The five BAPS clades are highlighted in different colours in the tree. The positions of the seven closed-genome strains are indicated by diamonds. Among the seven strains, four strains sequenced in this study are indicated by red diamonds. Bar, the mean number of nucleotide substitutions per site.

A total of 45 STs were identified in the O103:H2 strain set. While the majority of strains (*n*=2,169) belonged to ST17 and most of the remaining strains (*n*=489) belonged to the SLVs and DLVs of ST17 (32 and 6 STs, respectively), five STs were 4-locus variants (4LVs) and one was a 6-locus variant (6LV) of ST17 (Table 2). As the 4LV and the 6LV differed at five loci, this analysis revealed the presence of three distinct *E. coli* lineages with the O103:H2 serotype (Fig. 1a). Notably, none of the 4LV and 6LV strains (*n*=43) contained any of the marker genes of major STEC lineages (*stx, eae* and *ehxA*), except for one strain containing the *stx1c* gene alone. In the following analyses, we analysed the strains belonging to ST17 and its SLVs and DLVs (referred to as the major O103:H2 lineage; *n*=2,658). Among the 2,658 strains belonging to the major O103:H2 lineage, 2,169 and 369 strains belonged to ST17 and ST1967, respectively (Table S3). Examination of the distribution of major STEC virulence-related genes (*stx1*, *stx2*, *eae* and *ehxA*) revealed that while the *stx1a*, *eae* and *ehxA* genes were present in almost all strains, *stx2* was detected in a limited number of strains (Table 2).

### Phylogenetic and evolutionary overview of the major O103:H2 lineage

The 2658 strains belonging to the major O103:H2 lineage were isolated in 19 countries. We first constructed a WGS-based ML tree of these strains with the O26:H11 strain 11368 as an outlier and found that ST20 and its SLVs (ST1790 and ST9133; both were DLVs of ST17; Fig. 1a, Table S3) were first separated from the major O103:H2 lineage (Fig. S1). Therefore, we used the strains belonging to ST20 and its SLVs as outliers to construct the ML tree of the major O103:H2 lineage (Fig. 1b). Among the 2,658 genomes analysed, 249 were identical to one or more genomes in terms of their backbone chromosome sequence (88 groups; Table S1). To reduce strain redundancy, one representative strain was selected from each group and included in the following analyses. WGS-based phylogenetic analysis of this strain set (*n*=2497) revealed five distinct clades (C1–C5), as defined by Fastbaps-based clustering (Fig. 1b). Although the strain set included relatively small numbers of strains from nonhuman sources, these strains were distributed between human strains in each clade. The results of this phylogenetic analysis suggest that C1 (comprising mainly ST17 strains but including ST20 and other minor ST strains) was ancestral in the major O103:H2 lineage and that C2 (ST1967) and C3 (ST17, ST376 and ST386) emerged from C1, after which C4 (ST17) and C5 (ST17) emerged from C3. Notably, ST20 and its SLVs harboured neither *stx* nor *ehxA*. Although they contained the *eae* gene, its subtype (beta1) differed from that (epsilon1) of other strains in C1 and the other clades, indicating that the strains belonging to ST20 and its SLVs included in the strain set analysed in this study are EPEC and that an *eae* subtype change occurred upon the separation of the ST20 lineage from the other strains in the major O103:H2 lineage. Hereafter, strains other than the strains belonging to ST20 and its SLVs (*n*=2483) are referred to as STEC O103:H2.

Among the five clades, strains from the USA/Canada, showing the highest proportion in the current dataset (82%), presented the highest proportion in four of the clades (C1, C2, C4 and C5; 74–96%) (Fig. 1b; see Table S4 for details). However, most C3 strains (74%) were isolated in European countries (mainly from the UK but also other European countries; Table S4); thus, C3 is the clade circulating mainly in Europe. Analysis of the distribution of the major STEC virulence-related genes in the STEC O103:H2 strains revealed that while *stx1a*, *eae* (subtype epsilon1) and *ehxA* were well conserved, the *stx2a*, *stx2c* and *stx2d* genes were present in small numbers of strains (*n*=67, 3 and 15, respectively) (Fig. 1b, Table 2). Although the distributions of *stx2c* and *stx2d* were very limited to C1 and C3 (*stx2c*) and C4 (*stx2d*), *stx2a* was distributed in all clades but was distributed sporadically.

### General genomic features of STEC O103:H2 inferred from closed genomes

While three closed genome sequences are publicly available [9,38, 39], they belong to three of the five clades (C1, C4 and C5) (Table 3). To gain a wider genomic view of STEC O103:H2, particularly on Stx phages, the LEE and the STEC virulence plasmids, we determined the complete genome sequences of two strains belonging to C2 and C3, respectively, and two additional strains belonging to C4 and C5, respectively. As summarized in Table 3, the chromosomes of the seven strains were 5,259–5,580 kb in size. The chromosome backbone was well conserved between the strains, although many indels, such as the loss of Stx2a phage, were observed between the strains (Fig. S2). A small inversion occurred in the strains belonging to C3, C4 and C5 (Fig. S2). In particular, the chromosomes of the strain pairs belonging to the same clade were highly conserved (C4 and C5), even though the two C4 strains were distantly related in this clade. All strains carried an STEC virulence plasmid, and three strains contained one or two additional plasmids (see the next subsection for the details of these plasmids).

**Table 3.** General genomic features of the seven closed genomes

BAPS	C1	C2	C3	C4	C4	C5	C5
ST	ST17	ST1967	ST17	ST17	ST17	ST17	ST17
Strain	12009^†^	129^*^	PV16-126^*^	FWSEC0007^†^	CEC12044^*^	20151001^*^	2015 C-3163^*^
Accession no.	AP010958-9	AP038976-9	AP038987-8	CP031908-9	AP038983-6	AP038980-2	CP027219-20
Reference	[9]	This study	This study	[39]	This study	This study	[38]
Chromosome (kb)	5,449	5,580	5,259	5,398	5,458	5,555	5,500
CDSs^‡^	5,254	5,443	5,003	5,148	5,250	5,365	5,309
rRNAs operons	22	22	22	22	22	22	22
tRNAs	100	100	96	92	95	97	97
*stx* gene	1a/2a	1a×2	1a	1a	1a/2a	1a	1a
Plasmid (kb)	76^§^	73^§^/73/8	79^§^	73^§^	100/75^§^/2	94^§^/61	94^§^
CDSs	80	82/89/9	87	74	118/83/2	97/77	100
tRNAs	0	0/0/0	0	0	4/0/0	0/0	0
Total genome size (kb)	5,525	5,734	5,337	5,471	5,636	5,711	5,594

*Annotated using DFAST in this study.

†Reannotated using DFAST in this study.

‡CDSs, Predicted coding sequences.

§STEC virulence plasmid.

### Variation in Stx phages, the LEE and plasmids in the closed STEC O103:H2 genomes

#### Stx phages

**Fig. 2.**
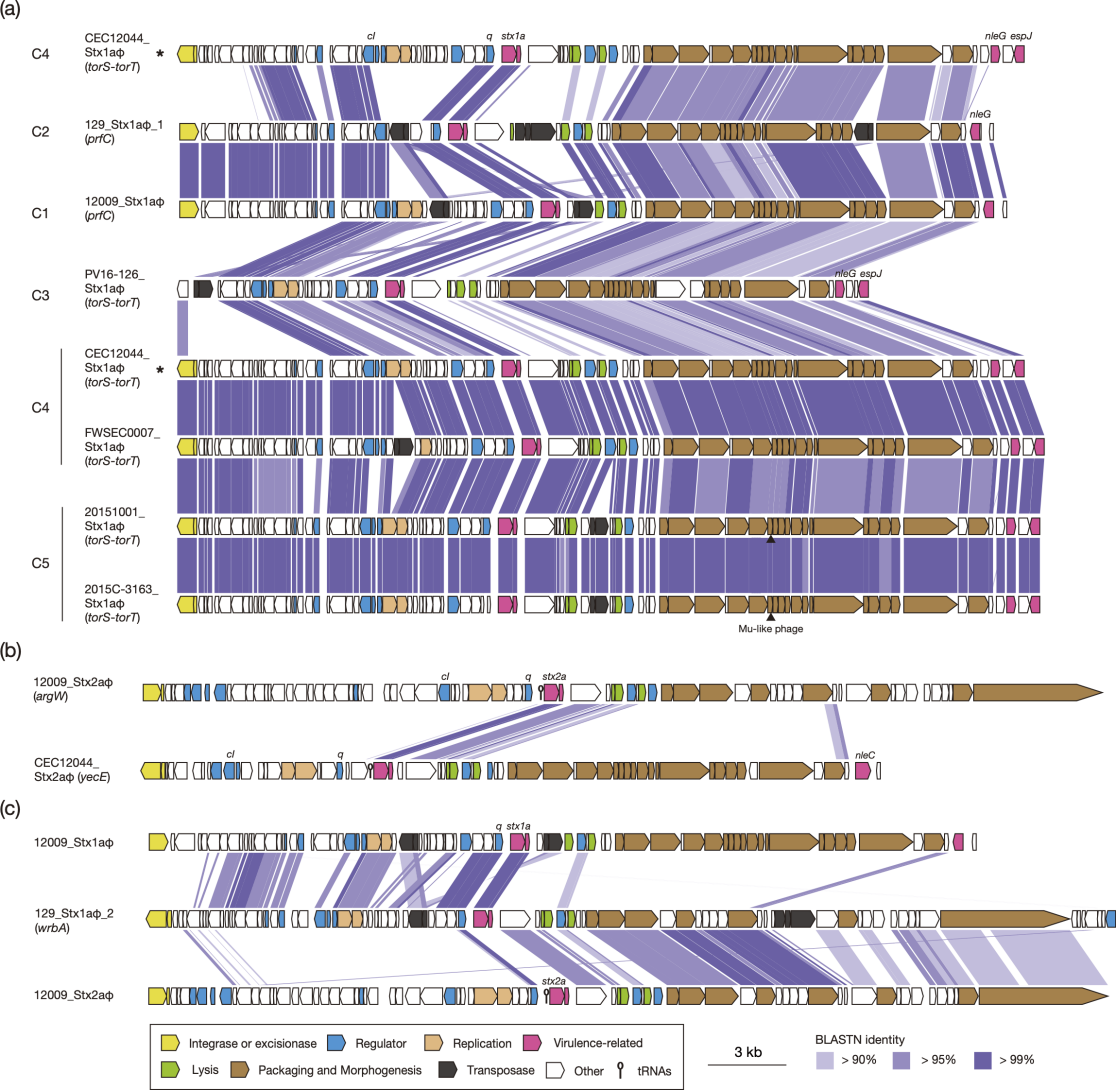
Comparison of the Stx phages identified in the seven closed-genome strains. Genomic organizations of seven long-tailed Stx1a phages (**a**), two Stx2a phages (**b**) and the short-tailed Stx1a phage of strain 129 and the long-tailed Stx1a phage and the short-tailed Stx2a phage of strain 12009 (**c**) are drawn at scale. The Stx1a phage of strain CEC12044, which is illustrated twice, is indicated by asterisks. The levels of nucleotide sequence identities between homologous coding sequences (CDSs) are indicated by heatmaps.

Of the seven strains with fully sequenced genomes (closed-genome strains), four contained an Stx1a phage alone, the strain belonging to C1 (strain 12009) and one of the two C4 strains (strain CEC12044) contained Stx1a and Stx2a phages and the strain belonging to C2 (strain 129) carried two Stx1a phages (Table 3). Among the eight Stx1a phages identified in the seven genomes, seven were long-tailed phages with a set of late genes similar to that of phage lambda (Fig. 2a), and the remaining phage, the second Stx1a phage in strain 129, was a short-tailed phage encoding a set of late genes similar to that of the Stx2a phages of O157:H7 STEC strains [40,41] (Fig. 2c).

The seven long-tailed Stx1a phages were 45.3–56.4 kb in size, but a transposable Mu-like phage (39.3 kb) [42] was integrated into the phages of both C5 strains (20151001 and 2015 C-3163) (Fig. 2a). Two loci (*prfC* and the *torS-torT* intergenic region) were identified as the integration sites of these Stx1a phages: the Stx1a phages of the C1 and C2 strains were found at *prfC*, and those of the C3, C4 and C5 strains were at *torS-torT*. While these seven Stx1a phage genomes presented overall sequence similarities, the sequences of the region including the *int* gene in the Stx1a phages of the C1 and C2 strains were different from those of the C4 and C5 strains. Although an IS-associated deletion occurred in this region in the C3 strain, a fragment of the *int* gene homologous to those of the C4 and C5 strains remained, suggesting that the Stx1a phages of the C3 strain originally contained the *int*-containing region similar to those of the C4 and C5 strains. Considering the phylogenetic relationship of the host strains, these findings suggest that genomic recombination inducing the integration site switch from *prfC* to *torS-torT* occurred in the Stx1a phage during the evolution of STEC O103:H2. Similar but more dynamic changes in Stx1a phages were observed in the O26:H11 ST21 lineage, including the turnover of Stx1a phage (replacement by apparently different Stx1a phages at the same or different chromosome loci) [43].

The sequences of the two Stx2a phages identified in the C1 and C4 strains were highly divergent: one was a long-tailed phage, and the other was a short-tailed phage (Fig. 2b). Interestingly, the second Stx1a phage of the C2 strain (strain 129) exhibited a chimeric structure with early and late regions similar to those of the long-tailed Stx1a phage and the short-tailed Stx2a phage of the C1 strain (strain 12009), respectively (Fig. 2c).

#### Locus of enterocyte effacement

LEE was present at *pheV* in all the closed-genome strains (Fig. S2). The core region encoding a set of T3SS genes was well conserved between the strains, although the integrase gene was degraded by the insertion of IS*629* in four strains (PV16-126, FWSEC0007, 20151001 and 2015 C-3163) (Fig. S3). These findings indicate that LEE is maintained at the *pheV* locus in the STEC O103:H2 lineage. However, various rearrangements, such as deletions and inversions, have occurred in the accessory region where several virulence-related genes, such as the *nleE*, *nleB* and *espL* genes for T3SS effectors, *ag43* (also known as *flu*) for the autotransporter antigen 43 [44] and *efa1* for adhesin Efa (*E. coli* factor for adherence; almost identical to lymphostatin encoded by *lifA*) [45], are encoded. Many of these rearrangements are also likely induced by IS-related mechanisms.

#### Plasmids

**Fig. 3.**
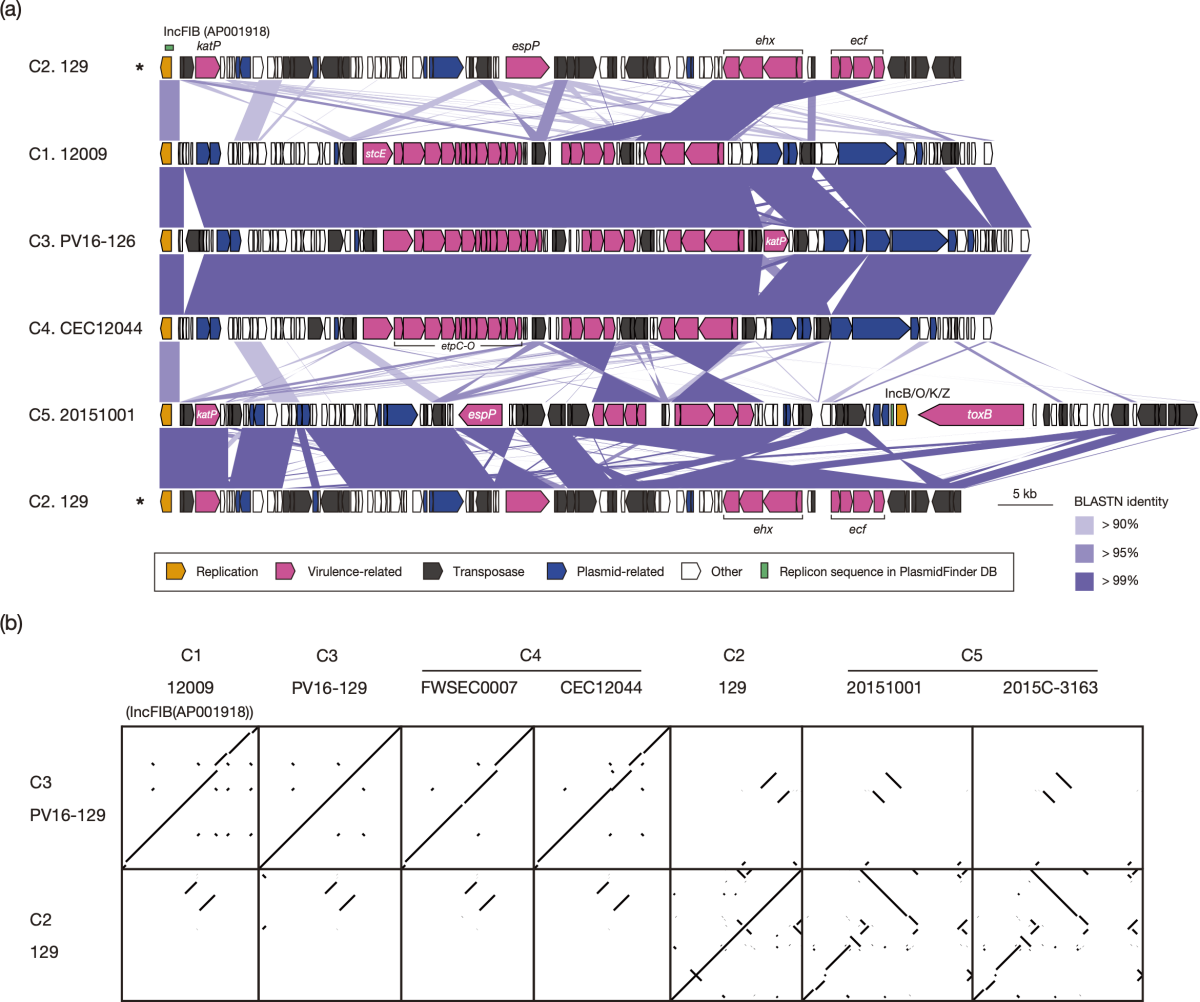
Variations in the STEC virulence plasmids among the seven closed-genome STEC O103:H2 strains. (**a**) Genomic organizations of the STEC virulence plasmids identified in the seven closed-genome strains. The levels of nucleotide sequence identity between homologous coding sequences (CDSs) are indicated by a heatmap. ‘Plasmid-related’ CDSs include genes for conjugation, mobilization, partitioning, maintenance and SOS inhibition. The plasmid of strain 129, which is illustrated twice, is indicated by asterisks. (**b**) Dot plot showing the sequence similarities (>99% sequence identity) between the virulence plasmids of the seven closed-genome strains. The nucleotide sequences of the virulence plasmids of strains PV16-129 (clade C3) and 129 (clade C2) were compared with those of other strains.

Virulence plasmids were highly conserved in sequence and gene organization between the C1, C3 and C4 strains (12009, PV16-126, FWSEC0007 and CEC12044), except for the insertion of the *katP* gene in the C3 strain and the insertion or deletion of a few IS elements (Fig. 3). However, the virulence plasmids of the C2 and C5 strains (129, 20151001 and 2015 C-3163) exhibited marked differences from those of the C1, C3 and C4 strains, and only the regions encoding the replication gene and the *ehx* and *ecf* operons were shared. Although the backbone sequences of the plasmids of the C2 and C5 strains were similar, marked structural variations due to IS-related deletions and inversions were observed between them. In addition, the *toxB* gene encoding an adhesin [46], which was first identified in the virulence plasmid of the O157:H7 strain Sakai (pO157) [47], was encoded by only the plasmids of the C5 strains. The two C5 plasmids contained the IncB/O/K/Z replicon in addition to the IncFIB (AP001918) replicon which was shared by all virulence plasmids (Fig. 3a). These results suggest that while the virulence plasmid acquired by the common ancestor of STEC O103:H2 has been maintained in C1, C3 and C4, it was replaced by other plasmids in C2 and C5. Although the virulence plasmids of the C2 and C5 strains presented some similarity, they were likely acquired independently.

The present genome assemblies inferred the presence of one or two additional plasmids in strains 129 (C2), CEC12044 (C4) and 20151001 (C5) (Fig. S3a). Of these, two plasmids (72.8 and 61.4 kb) carried by strains 129 and 20151001 shared a set of genes for conjugation, stabilization and SOS inhibition, but antimicrobial resistance genes, the streptomycin resistance genes (*strAB*), the sulphonamide resistance gene (*sul*) and the TEM-1 *β*-lactamase gene (*bla*_TEM-1B_), were found only in the plasmid of strain 129. For the large plasmid (101 kb) carried by strain CEC12044, the functions of most genes are unknown, except for several genes, such as three prophage-related genes, a DNA polymerase theta subunit-encoding gene and two tRNA genes.

### Variation in the plasmid-encoded virulence-related gene repertoire among the STEC O103:H2 strains

As the virulence-related genes of the virulence plasmids of the closed-genome strains showed notable variation, we analysed the distribution of six plasmid-encoded virulence-related genes in the major O103:H2 lineage (Fig. 1b; note that the ST20 strains and their close relatives lacked not only *ehxA* but also the other five genes). Among the six genes, *ehxA* and *ecf1*, which were found in the virulence plasmids of all the closed-genome strains, were well conserved in the entire STEC O103:H2 lineage. High conservation of these genes has also been observed in other STEC lineages, such as STEC O26:H11, O121:H19 and O145:H28, as well as in STEC belonging to clonal complex 119 (CC119; O165:H25 and O172:H25) [24,34, 37, 48], suggesting the biological importance of these two genes (or the *ehx* and *ecf* operons) in STEC. The results of the analysis of STEC O103:H2 obtained in this study provide further support for this notion. The remaining four genes (*stcE*, *katP*, *espP* and *toxB*) presented distribution patterns unique to each clade. In C1, while strains belonging to early separated branches contained *espP* and *toxB* or *espP* alone, close relatives of strain 12009 contained only the *stcE* gene, as observed for this closed-genome strain. In C2, most strains contained *katP* and *espP*, as observed for the closed-genome strain (129). In C3, most strains contained *stcE* and *katP*, as observed for the closed-genome strains (PV16-126), but strains belonging to one sublineage (ST386 strains) contained *katP*, *espP* and *toxB*. In C4, while *katP* and *espP* were sporadically found in several strains, most strains contained only *stcE*, as observed for the closed-genome strains (FWSEC0007 and CEC12044). In C5, most strains contained *katP*, *espP* and *toxB,* as observed for the closed-genome strains (201510001 and 2015 C-3168), but several strains that were separated early from the other strains in clade C5 contained *stcE* and *katP*. These findings suggest that the repertoires of virulence-related genes on the virulence plasmids in each clade were largely consistent with those identified in the closed-genome strains belonging to each clade, but there were considerable intraclade variations. The abundance of IS elements on the virulence plasmid genomes may be related to the generation of such intraclade variations.

## Conclusion

In this study, we analysed the WGS data for 2701 O103:H2 strains isolated from various geographic regions, including the 193 Japanese strains sequenced in this study. These strains belong to three distinct *E. coli* lineages, and the virulence marker genes for typical STEC (*eae*, *stx* and *ehxA*) are found only in the major lineage (ST17 and its SLVs and DLVs; called the major O103:H2 lineage in this manuscript). We defined five clades (C1-C5) in the major O103:H2 lineage, of which C1 was the ancestral clade, C2 and C3 emerged from C1 and C4 and C5 emerged from C3. As ST20 and its SLVs found in clade C1 did not contain *stx* and *ehxA* and the subtype of their *eae* genes was different from that of the other strains, the globally circulating STEC O103:H2 lineage emerged by acquiring *stx* and *ehxA* through the acquisition of phages and plasmids encoding these genes after separating from ST20 and its SLVs. A change in the *eae* subtype also occurred in this process. While *stx2a*, *stx2c* and *stx2d* were sporadically distributed in limited STEC O103:H2 strains, *stx1*, *eae* (epsilon1 subtype) and *ehxA* were highly conserved in the entire STEC O103:H2 lineage. However, detailed analyses of the closed genomes of seven STEC O103:H2 strains covering the five clades revealed marked variations in the genetic elements encoding these genes, such as rearrangements in the LEE accessory region and a shift in the integration sites of the long-tailed Stx1a phage due to the replacement of the *int*-containing genomic segments. Marked genomic diversity was also observed for the virulence plasmids, which were generated by the replacement of plasmids and the gain and loss of virulence-related genes, the latter of which was likely related to IS elements that abundantly occur in virulence plasmids. These results provide the current global phylogenetic overview of O103:H2 strains and expand our understanding of the variation in the major virulence determinants within STEC O103:H2, which is relatively understudied among the major STEC lineages.

## Supplementary material

10.1099/mgen.0.001625 Uncited Supplementary Material 1.

10.1099/mgen.0.001625 Uncited Supplementary Material 2.

## Abbreviations

CC17: clonal complex 17
DLVs: double-locus variants
EPEC: enteropathogenic *Escherichia coli*
IE: integrative element
IS: insertion sequence
LEE: locus of enterocyte effacement
ML: maximum likelihood
MST: minimum spanning tree
ONT: Oxford Nanopore Technologies
PP: prophage
SLVs: single-locus variants
ST: sequence type
STEC: Shiga toxin-producing *Escherichia coli*
Stx: Shiga toxin
T3SS: type III secretion system
WGS: whole-genome sequence

## References

[1] Ylinen E, Salmenlinna S, Halkilahti J, Jahnukainen T, Korhonen L (2020). Hemolytic uremic syndrome caused by Shiga toxin-producing *Escherichia coli* in children: incidence, risk factors, and clinical outcome. Pediatr Nephrol.

[2] Dato L, Mancuso MC, Daprai L, Ria T, Rossetti D (2025). Bloody diarrhea, STEC infection, and HUS in the molecular microbiology era. Pediatr Nephrol.

[3] Scheutz F, Teel LD, Beutin L, Piérard D, Buvens G (2012). Multicenter evaluation of a sequence-based protocol for subtyping Shiga toxins and standardizing Stx nomenclature. J Clin Microbiol.

[4] Probert WS, McQuaid C, Schrader K (2014). Isolation and identification of an *Enterobacter cloacae* strain producing a novel subtype of Shiga toxin type 1. J Clin Microbiol.

[5] Lindsey RL, Prasad A, Feldgarden M, Gonzalez-Escalona N, Kapsak C (2023). Identification and characterization of ten *Escherichia coli* strains encoding novel shiga toxin 2 Subtypes, Stx2n as well as Stx2j, Stx2m, and Stx2o, in the United States. Microorganisms.

[6] Croxen MA, Law RJ, Scholz R, Keeney KM, Wlodarska M (2013). Recent advances in understanding enteric pathogenic *Escherichia coli*. Clin Microbiol Rev.

[7] Deng W, Puente JL, Gruenheid S, Li Y, Vallance BA (2004). Dissecting virulence: systematic and functional analyses of a pathogenicity island. Proc Natl Acad Sci USA.

[8] Tobe T, Beatson SA, Taniguchi H, Abe H, Bailey CM (2006). An extensive repertoire of type III secretion effectors in *Escherichia coli* O157 and the role of lambdoid phages in their dissemination. Proc Natl Acad Sci USA.

[9] Ogura Y, Ooka T, Iguchi A, Toh H, Asadulghani M (2009). Comparative genomics reveal the mechanism of the parallel evolution of O157 and non-O157 enterohemorrhagic *Escherichia coli*. Proc Natl Acad Sci USA.

[10] Nemati A, Askari Badouei M, Hashemitabar G, Hafiz M (2025). Shiga toxin-producing *Escherichia coli* plasmid diversity reveals virulence potential and control opportunities in animal hosts. Sci Rep.

[11] Mariani-Kurkdjian P, Denamur E, Milon A, Picard B, Cave H (1993). Identification of a clone of *Escherichia coli* O103:H2 as a potential agent of hemolytic-uremic syndrome in France. J Clin Microbiol.

[12] Tarr PI, Fouser LS, Stapleton AE, Wilson RA, Kim HH (1996). Hemolytic–uremic syndrome in a six-year-old girl after a urinary tract infection with Shiga-toxin–producing *Escherichia coli* O103:H2. N Engl J Med.

[13] Vishram B, Jenkins C, Greig DR, Godbole G, Carroll K (2021). The emerging importance of Shiga toxin-producing *Escherichia coli* other than serogroup O157 in England. J Med Microbiol.

[14] Karama M, Johnson RP, Holtslander R, Gyles CL (2008). Phenotypic and genotypic characterization of verotoxin-producing *Escherichia coli* O103:H2 isolates from cattle and humans. J Clin Microbiol.

[15] Bibbal D, Loukiadis E, Kérourédan M, Ferré F, Dilasser F (2015). Prevalence of carriage of Shiga toxin-producing *Escherichia coli* serotypes O157:H7, O26:H11, O103:H2, O111:H8, and O145:H28 among slaughtered adult cattle in France. Appl Environ Microbiol.

[16] Tack DM, Kisselburgh HM, Richardson LC, Geissler A, Griffin PM (2021). Shiga toxin-producing *Escherichia coli* outbreaks in the United States, 2010-2017. Microorganisms.

[17] Brooks JT, Sowers EG, Wells JG, Greene KD, Griffin PM (2005). Non-O157 Shiga toxin-producing *Escherichia coli* infections in the United States, 1983-2002. J Infect Dis.

[18] Iguchi A, Iyoda S, Ohnishi M, EHEC Study Group (2012). Molecular characterization reveals three distinct clonal groups among clinical shiga toxin-producing *Escherichia coli* strains of serogroup O103. J Clin Microbiol.

[19] Poh C-YJ, Rodwell EV, Godbole G, Jenkins C (2024). Genotypic analysis of Shiga toxin-producing *Escherichia coli* clonal complex 17 in England and Wales, 2014-2022. J Med Microbiol.

[20] Parks DH, Imelfort M, Skennerton CT, Hugenholtz P, Tyson GW (2015). CheckM: assessing the quality of microbial genomes recovered from isolates, single cells, and metagenomes. Genome Res.

[21] Ingle DJ, Valcanis M, Kuzevski A, Tauschek M, Inouye M (2016). *In silico* serotyping of *E. coli* from short read data identifies limited novel O-loci but extensive diversity of O:H serotype combinations within and between pathogenic lineages. Microb Genom.

[22] Inouye M, Dashnow H, Raven L-A, Schultz MB, Pope BJ (2014). SRST2: rapid genomic surveillance for public health and hospital microbiology labs. Genome Med.

[23] Zhou Z, Alikhan N-F, Sergeant MJ, Luhmann N, Vaz C (2018). GrapeTree: visualization of core genomic relationships among 100,000 bacterial pathogens. Genome Res.

[24] Ogura Y, Gotoh Y, Itoh T, Sato MP, Seto K (2017). Population structure of *Escherichia coli* O26 : H11 with recent and repeated *stx2* acquisition in multiple lineages. Microb Genom.

[25] Wick RR, Judd LM, Gorrie CL, Holt KE (2017). Completing bacterial genome assemblies with multiplex MinION sequencing. Microb Genom.

[26] De Coster W, D’Hert S, Schultz DT, Cruts M, Van Broeckhoven C (2018). NanoPack: visualizing and processing long-read sequencing data. Bioinformatics.

[27] Murigneux V, Roberts LW, Forde BM, Phan M-D, Nhu NTK (2021). MicroPIPE: validating an end-to-end workflow for high-quality complete bacterial genome construction. BMC Genomics.

[28] Wick RR, Judd LM, Gorrie CL, Holt KE (2017). Unicycler: resolving bacterial genome assemblies from short and long sequencing reads. PLoS Comput Biol.

[29] Tanizawa Y, Fujisawa T, Nakamura Y (2018). DFAST: a flexible prokaryotic genome annotation pipeline for faster genome publication. Bioinformatics.

[30] Ohtsubo Y, Ikeda-Ohtsubo W, Nagata Y, Tsuda M (2008). GenomeMatcher: a graphical user interface for DNA sequence comparison. BMC Bioinformatics.

[31] Carattoli A, Zankari E, García-Fernández A, Voldby Larsen M, Lund O (2014). *In silico* detection and typing of plasmids using PlasmidFinder and plasmid multilocus sequence typing. Antimicrob Agents Chemother.

[32] Kurtz S, Phillippy A, Delcher AL, Smoot M, Shumway M (2004). Versatile and open software for comparing large genomes. Genome Biol.

[33] Croucher NJ, Page AJ, Connor TR, Delaney AJ, Keane JA (2015). Rapid phylogenetic analysis of large samples of recombinant bacterial whole genome sequences using Gubbins. Nucleic Acids Res.

[34] Nishida R, Nakamura K, Taniguchi I, Murase K, Ooka T (2021). The global population structure and evolutionary history of the acquisition of major virulence factor-encoding genetic elements in Shiga toxin-producing *Escherichia coli* O121:H19. Microb Genom.

[35] Tonkin-Hill G, Lees JA, Bentley SD, Frost SDW, Corander J (2019). Fast hierarchical Bayesian analysis of population structure. Nucleic Acids Res.

[36] Letunic I, Bork P (2024). Interactive Tree of Life (iTOL) v6: recent updates to the phylogenetic tree display and annotation tool. Nucleic Acids Res.

[37] Nakamura K, Seto K, Lee K, Ooka T, Gotoh Y (2023). Global population structure, genomic diversity and carbohydrate fermentation characteristics of clonal complex 119 (CC119), an understudied Shiga toxin-producing *E. coli* (STEC) lineage including O165:H25 and O172:H25. *Microbial Genomics*.

[38] Patel PN, Lindsey RL, Garcia-Toledo L, Rowe LA, Batra D (2018). High-Quality whole-genome sequences for 77 Shiga toxin-producing *Escherichia coli* strains generated with PacBio sequencing. Genome Announc.

[39] Tyson S, Peterson C-L, Olson A, Tyler S, Knox N (2019). Eleven high-quality reference genome sequences and 360 draft assemblies of Shiga toxin-producing *Escherichia coli* isolates from human, food, animal, and environmental sources in Canada. Microbiol Resour Announc.

[40] Plunkett G, Rose DJ, Durfee TJ, Blattner FR (1999). Sequence of Shiga toxin 2 Phage 933W from *Escherichia coli* O157:H7: Shiga toxin as a phage late-gene product. J Bacteriol.

[41] Asadulghani M, Ogura Y, Ooka T, Itoh T, Sawaguchi A (2009). The defective prophage pool of *Escherichia coli* O157: prophage-prophage interactions potentiate horizontal transfer of virulence determinants. PLoS Pathog.

[42] Harshey RM (2014). Transposable Phage Mu. Microbiol Spectr.

[43] Yano B, Taniguchi I, Gotoh Y, Hayashi T, Nakamura K (2023). Dynamic changes in Shiga toxin (Stx) 1 transducing phage throughout the evolution of O26:H11 Stx-producing *Escherichia coli*. Sci Rep.

[44] Ulett GC, Valle J, Beloin C, Sherlock O, Ghigo J-M (2007). Functional analysis of antigen 43 in uropathogenic *Escherichia coli* reveals a role in long-term persistence in the urinary tract. Infect Immun.

[45] Stevens MP, van Diemen PM, Frankel G, Phillips AD, Wallis TS (2002). Efa1 influences colonization of the bovine intestine by shiga toxin-producing *Escherichia coli* serotypes O5 and O111. Infect Immun.

[46] Tatsuno I, Horie M, Abe H, Miki T, Makino K (2001). *toxB* gene on pO157 of enterohemorrhagic *Escherichia coli* O157:H7 is required for full epithelial cell adherence phenotype. Infect Immun.

[47] Makino K, Ishii K, Yasunaga T, Hattori M, Yokoyama K (1998). Complete nucleotide sequences of 93-kb and 3.3-kb plasmids of an enterohemorrhagic *Escherichia coli* O157:H7 derived from Sakai outbreak. DNA Res.

[48] Nakamura K, Murase K, Sato MP, Toyoda A, Itoh T (2020). Differential dynamics and impacts of prophages and plasmids on the pangenome and virulence factor repertoires of Shiga toxin-producing *Escherichia coli* O145:H28. Microb Genom.

