# Erratum: Global population structure of Shiga toxin-producing Escherichia coli O103:H2 and the variation in their major virulence factor-encoding genetic elements

**DOI:** 10.1099/mgen.0.001661

**Published:** 2026-03-02

**Authors:** Itsuki Taniguchi, Yo Morimoto, Yoko Kimura, Junji Seto, Yuko Kawai, Tomoko Kitahashi, Junko Aoki, Katsuya Terai, Toshihiko Furuta, Yuki Wakabayashi, Sumiko Tanabe, Mitsuhiro Hamasaki, Yuri Abe, Mari Sasaki, Hiroshi Narimatsu, Eiji Yokoyama, Sunao Iyoda, Tetsuya Hayashi, Keiji Nakamura

**Affiliations:** 1 Graduate School of Medical Sciences, Kyushu University, Fukuoka, Japan; 2 Hokkaido Institute of Public Health, Sapporo, Japan; 3 Miyagi Prefectural Institute of Public Health and Environment, Sendai, Japan; 4 Yamagata Prefectural Institute of Public Health, Yamagata, Japan; 5 Gunma Prefectural Institute of Public Health and Environmental Sciences, Gunma, Japan; 6 Chiba City Institute of Health and Environment, Chiba, Japan; 7 Niigata Prefectural Institute of Public Health and Environmental Sciences, Niigata, Japan; 8 Shizuoka Institute of Environment and Hygiene, Shizuoka, Japan; 9 Hamamatsu City Health Environment Research Center, Shizuoka, Japan; 10 Osaka Institute of Public Health, Osaka, Japan; 11 Nara Prefecture Institute of Health, Nara, Japan; 12 Fukuoka Institute of Health and Environmental Sciences, Fukuoka, Japan; 13 Fukuoka City Institute of Health and Environment, Fukuoka, Japan; 14 Oita Prefectural Institute of Health and Environment, Oita, Japan; 15 Chiba Prefectural Institute of Public Health, Chiba, Japan; 16 National Institute of Infectious Diseases, Tokyo, Japan

In the published version of this article, an error was identified in the Funding Information section. One of the grant numbers was incorrectly listed as 25fk0108702s070 instead of 25fk0108702s0702. This has now been corrected in the original article.

The Microbiology Society apologises for any inconvenience caused.

