# Supplementary material for: Global population structure of Shiga toxin-producing Escherichia coli O103:H2 and the variation in their major virulence factor-encoding genetic elements
Source: Microb Genom. 2026 Jan 23;12(1):001625. doi: 10.1099/mgen.0.001625 (PMC12831630; doi:10.1099/mgen.0.001625)
Supplement: Uncited Supplementary Material 1. [file mgen-12-01625-s001.pdf]

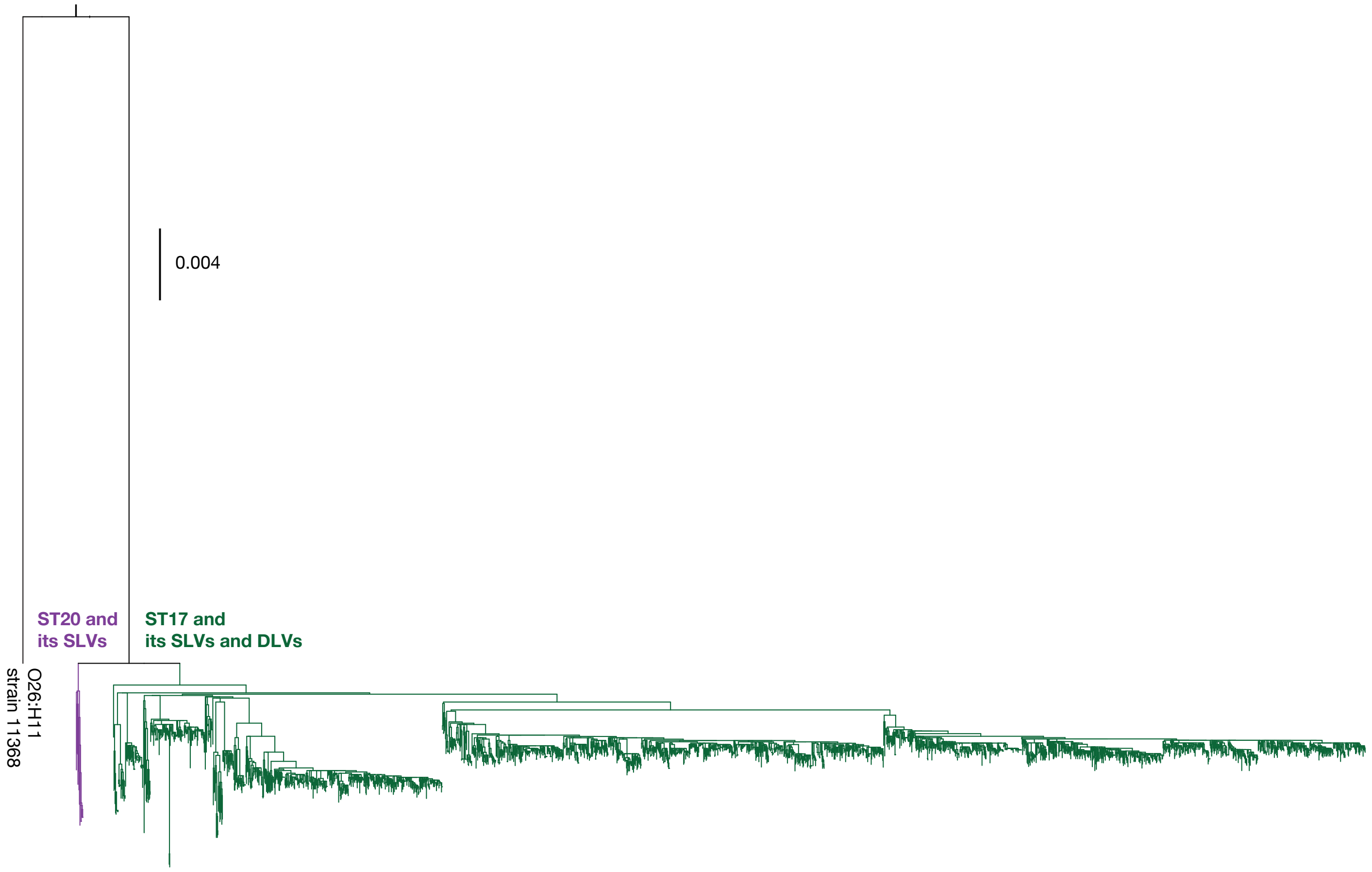

Fig. S1 Phylogenetic relationships of the strains belonging to ST20 and its SLV strains with other strains belonging to the major O103:H2 lineage.

An ML tree was constructed on the basis of 13,710 SNPs identified on the PP/IE/IS-free and recombination-free chromosomal backbone (1,755,366 bp), with the O26:H11 strain 11368 as an outlier.

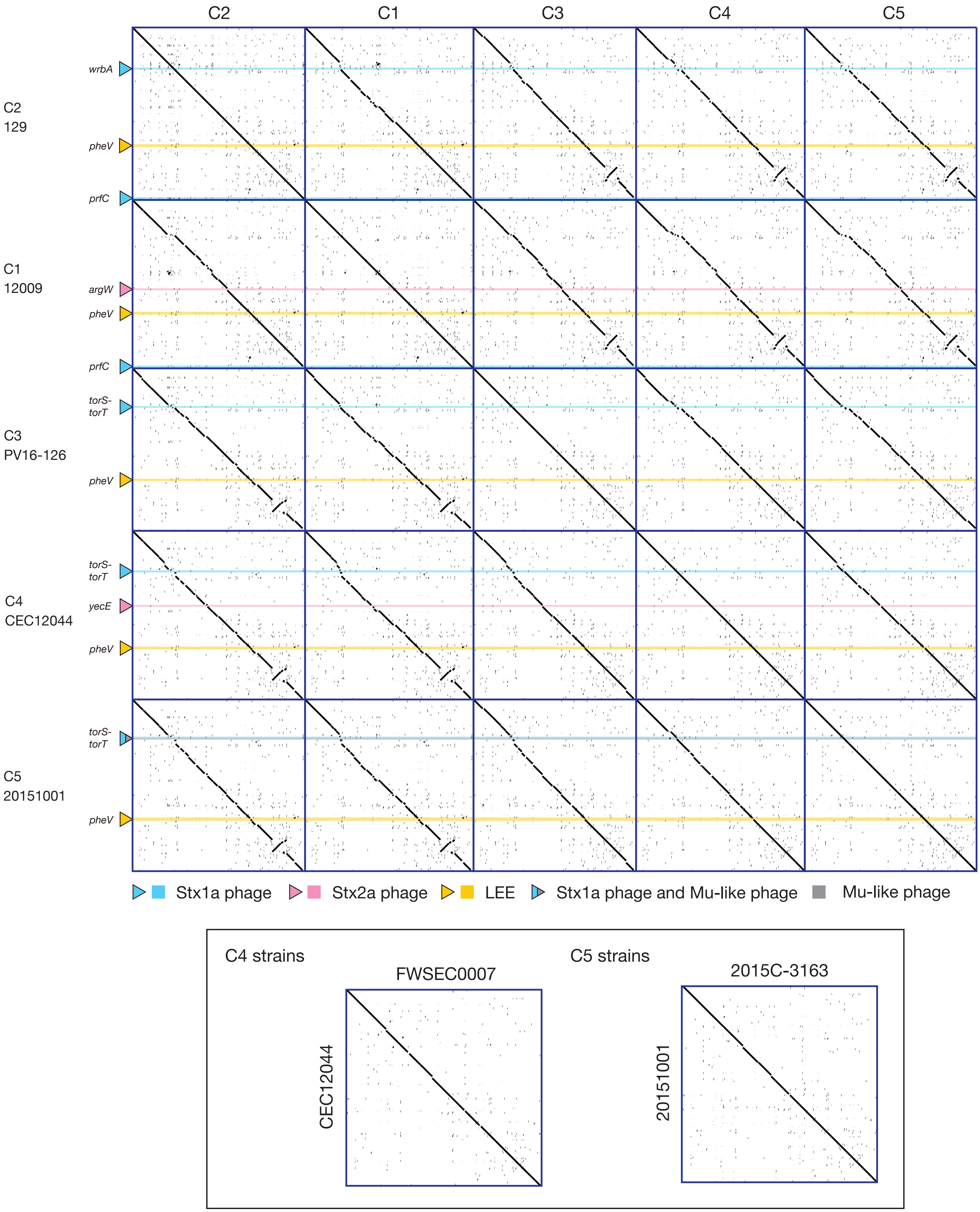

Fig. S2 Comparison of the chromosome sequences of closed genomes.

Dot plot matrices of chromosome sequences between five closed genomes are shown (of the two strains in clades C4 and C5, only one strain is shown for each clade). Only the sequences with >99% identity are shown. The positions of Stx phages and LEEs are indicated. Dot plot matrices of the chromosome sequences between two strains belonging to the same clade (C4 and C5) are shown in the inset.

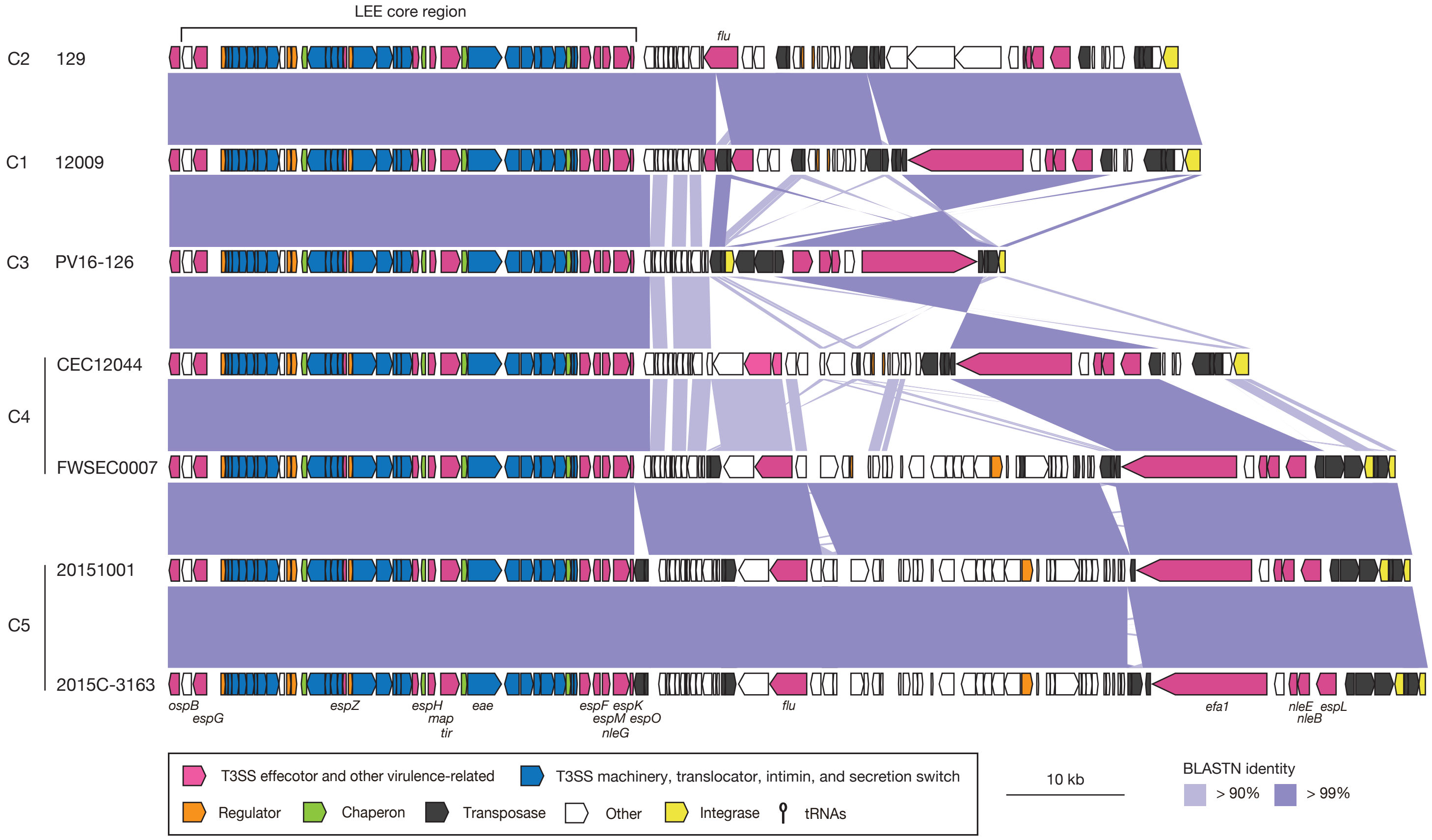

Fig. S3 Comparison of the LEE elements from the seven closed-genome strains.

The genetic structures of the LEE elements of the seven strains are drawn to scale. The levels of nucleotide sequence identities are indicated by a heatmap.

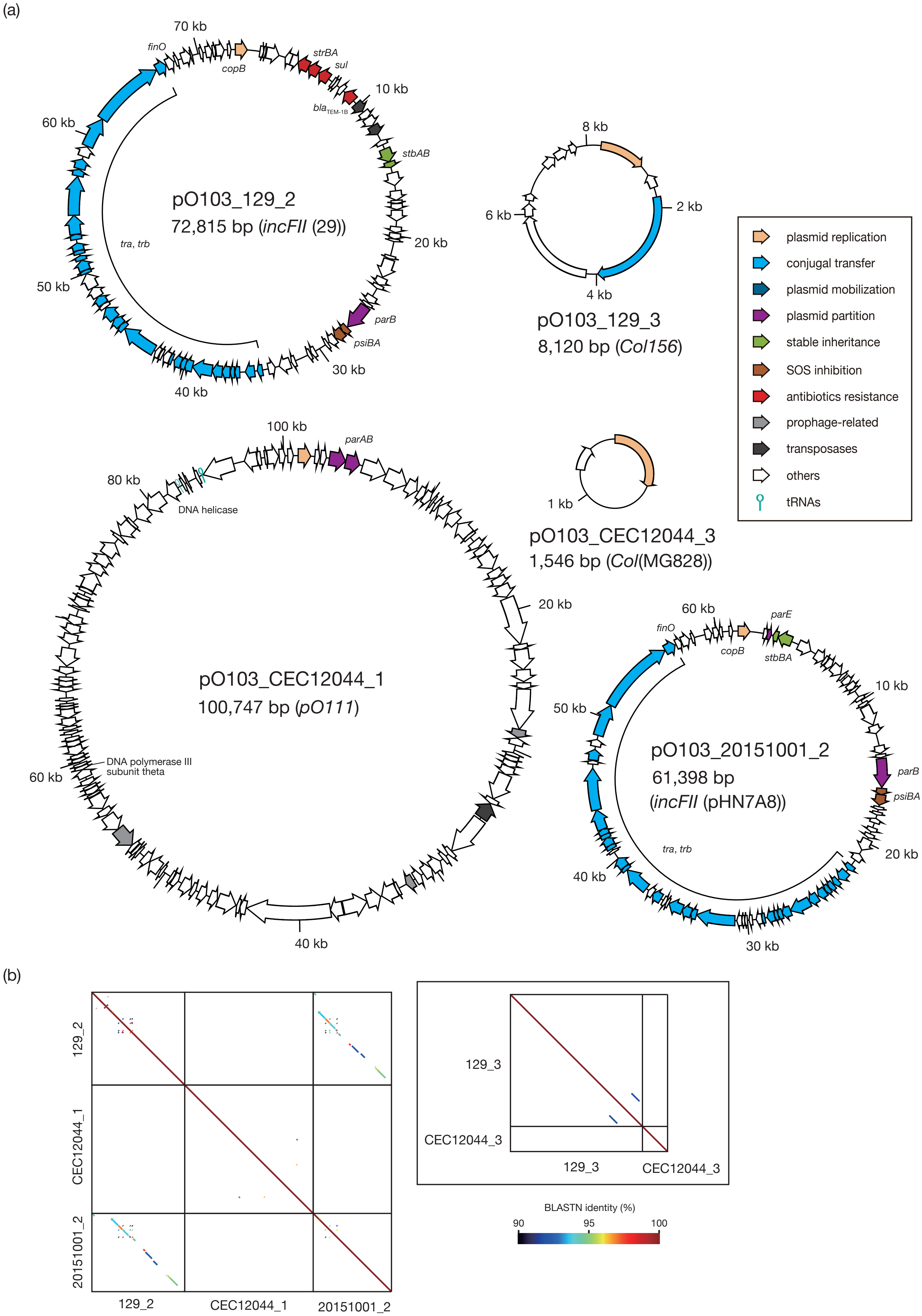
(a)
pO103_129_2
72,815 bp (incFII (29))
70 kb
10 kb
20 kb
30 kb
40 kb
50 kb
60 kb
finO
copB
strBA
sul
blaTEM-1B
stbAB
parB
psiBA
tra, trb
pO103_129_3
8,120 bp (Col156)
8 kb
2 kb
4 kb
6 kb
plasmid replication
conjugal transfer
plasmid mobilization
plasmid partition
stable inheritance
SOS inhibition
antibiotics resistance
prophage-related
transposases
others
tRNAs
pO103_CEC12044_1
100,747 bp (pO111)
100 kb
parAB
80 kb
DNA helicase
20 kb
DNA polymerase III subunit theta
60 kb
40 kb
pO103_CEC12044_3
1,546 bp (Col(MG828))
1 kb
pO103_20151001_2
61,398 bp
(incFII (pHN7A8))
60 kb
finO
parE
copB
stbBA
10 kb
50 kb
parB
psiBA
tra, trb
20 kb
40 kb
30 kb
(b)
129_2
CEC12044_1
20151001_2
129_2
CEC12044_1
20151001_2
129_3
CEC12044_3
129_3
CEC12044_3
BLASTN identity (%)
90
95
100

Fig. S4 Nonvirulence plasmids identified in the three closed-genome strains.

(a) Circular maps of five nonvirulence plasmids. Replicons identified by PlasmidFinder 2.1 (https://cge.food.dtu.dk/services/PlasmidFinder/) are shown in parentheses. (b) Dot plot analyses of the three nonvirulence plasmids (other than two small plasmids) to show their nucleotide sequence similarity (>90% identity). The sequence similarity between two small plasmids is separately shown in the inset.
